# Protein Binding of Benzofuran Derivatives: A CD Spectroscopic and In Silico Comparative Study of the Effects of 4-Nitrophenyl Functionalized Benzofurans and Benzodifurans on BSA Protein Structure

**DOI:** 10.3390/biom12020262

**Published:** 2022-02-05

**Authors:** Pasqualina Liana Scognamiglio, Caterina Vicidomini, Francesco Fontanella, Claudio De Stefano, Rosanna Palumbo, Giovanni N. Roviello

**Affiliations:** 1 Center for Advanced Biomaterial for Health Care (CABHC), Istituto Italiano di Tecnologia, I-80125 Naples, Italy; pasqualina.scognamiglio@iit.it; 2 Istituto di Biostrutture e Bioimmagini IBB-CNR, via Tommaso De Amicis 95, I-80145 Naples, Italy; caterina.vicidomini@ibb.cnr.it (C.V.); rosanna.palumbo@cnr.it (R.P.); 3 Department of Electrical and Information Engineering (DIEI), University of Cassino and Southern Lazio, 03043 Cassino (FR), Italy; fontanella@unicas.it (F.F.); destefano@unicas.it (C.D.S.)

**Keywords:** serum albumin, benzofuran ligands, circular dichroism, fluorescence titration, molecular docking, protein–ligand interactions

## Abstract

Benzofuran derivatives are synthetic compounds that are finding an increasing interest in the scientific community not only as building blocks for the realization of new materials, but also as potential drugs thanks to their ability to interact with nucleic acids, interfere with the amyloid peptide aggregation and cancer cell cycle. However, their ability to interact with proteins is a theme still in need of investigation for the therapeutic importance that benzofurans could have in the modulation of protein-driven processes and for the possibility of making use of serum albumins as benzofurans delivery systems. To this scope, we investigated the protein binding ability of two 4-nitrophenyl-functionalized benzofurans previously synthesized in our laboratory and herein indicated as BF1 and BDF1, which differed for the number of furan rings (a single moiety in BF1, two in BDF1), using bovine serum albumin (BSA) as a model protein. By circular dichroism (CD) spectroscopy we demonstrated the ability of the two heteroaromatic compounds to alter the secondary structure of the serum albumin leading to different consequences in terms of BSA thermal stability with respect to the unbound protein (ΔT_m_ > 3 °C for BF1, −0.8 °C for BDF1 with respect to unbound BSA, in PBS buffer, pH 7.5) as revealed in our CD melting studies. Moreover, a molecular docking study allowed us to compare the possible ligand binding modes of the mono and difuranic derivatives showing that while BF1 is preferentially housed in the interior of protein structure, BDF1 is predicted to bind the albumin surface with a lower affinity than BF1. Interestingly, the different affinity for the protein target predicted computationally was confirmed also experimentally by fluorescence spectroscopy (k_D_ = 142.4 ± 64.6 nM for BDF1 vs. 28.4 ± 10.1 nM for BF1). Overall, the above findings suggest the ability of benzofurans to bind serum albumins that could act as their carriers in drug delivery applications.

## 1. Introduction

Albumins are proteins with high peptide sequences homology, with bovine and human albumins sharing 76% identity [1], abundant in the circulatory system of mammals where they contribute significantly to the osmotic blood pressure [2]. Albumins, and primarily BSA, are typically used as protein models and their binding with molecules proposed for biotechnological applications is investigated very extensively in both academic and industrial research areas [2,3,4,5,6,7,8,9]. Structurally, albumins present two domains with at least two high-affinity binding sites, as well as other various low-affinity ones [10].

The major physiological function of albumins is the transport of many classes of ligands, including cations, fatty acids, steroids, and amino acids present in the bloodstream to their target organs [1,11]. Interestingly, this binding has also driven the pharmaceutical use of albumins as drug carriers [12,13,14].

Albumins are quite soluble, but frequent encounters between their molecules lead to their aggregation, as ascertained for example with BSA [15] leading to dimers and higher aggregates [16,17], a major factor in protein function [18] and stability [19,20]. More in detail, electrophoretic analysis in nondenaturing gels revealed that the monomer/dimer ratio of BSA is higher than 80% [21], but the albumin oligomerization state can be influenced by the interactions with ligands that, as observed in the case of myristic acid, when ligated to the dimers make them less stable and more prone to dissociate into monomers [22]. Interestingly, albumin oligomerization leads to substantial amounts of β-sheet structures which are directly correlated with aggregation [22,23], as well as a thermal stabilization (by more than 3 °C) of the serum albumin in the dimeric form with respect to the monomer [24].

Oxygen-containing heterocycles are an important class of molecules that exhibit interesting biological and therapeutic activities and share structural similarity with several natural bioactive compounds [25,26,27]. Among these, benzofurans have gained a considerable interest being endowed with a wide range of biological activities such as antibiotic [28,29,30], anti-inflammatory [31], anti-parasitic [32], anticancer [33,34,35], neuroprotective and analgesic [36] effects. As for the molecular basis of the anticancer activity of benzofurans, the nucleic acid binding [37], as well as the inhibition of particular serine/threonine kinases involved in tumour development, and cancer cell cycle modifications are some of the proposed mechanisms [34,38].

**Figure 1:**
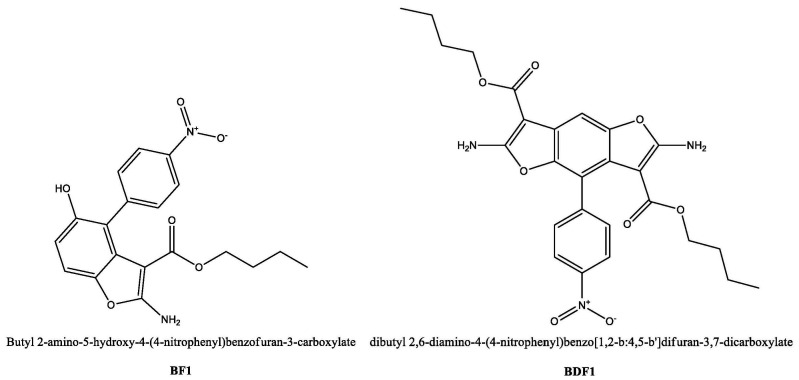
Chemical structures for the compounds studied as protein ligands in the current work.

Some of us have recently investigated the biological properties of compounds containing 4-nitrophenyl-functionalized benzofuran (BF) and benzodifuran (BDF) moieties, finding that both classes were endowed with antiproliferative activity on prostatic tumour cells (PC-3) in direct correlation with the lipophilicity of the heterocycles, with the compounds denominated BDF1 and especially BF1 (Figure 1) being the most active candidates [38]. As a prosecution of that previous work, we decided to investigate the ability of both 4-nitrophenyl-functionalized benzofuran and benzodifuran derivatives to interact with proteins, using as a model bovine serum albumin (BSA), for a better comprehension of the analogies and differences that the heteroaromatic derivatives display with respect to the binding with this fundamental family of biomacromolecules involved in numerous therapeutically relevant pathways.

## 2. Materials and Methods

### 2.1. Synthesis of Benzofuran Derivatives BF1 and BDF1

The benzodifurans used in this work were synthesized according to a procedure described in the literature based on the Craven reaction [38]. All the intermediates used in the synthetic procedures have been purchased by Sigma Aldrich and Acros Organics, and used without further purification. The purity of BF1, and BDF1 was ≥95%.

### 2.2. CD Binding Studies

Circular dichroism (CD) spectra were registered on a Jasco J-715 spectropolarimeter equipped with a Peltier PTC-423S/15 in a Hellma quartz cell with a light path of 0.1 cm according to other previous literature experiments [39,40,41,42,43]. All the spectra were averaged over 3 scans. All experiments were performed on solutions buffered at pH 7.4. The concentration of BSA (Sigma) was 0.12 μM, while the benzofuran stock solutions were 80 mM in DMSO.

### 2.3. CD Denaturation Studies

CD denaturation experiments on BSA/benzofuran complexes were realized recording the ellipticity at 222 nm with a temperature scan rate of 1 °C/min in the range 50–90 °C. All curve data were normalised and smoothed within the given range and the first derivatives of the melting curves were calculated. Therefore, T_m_ value was determined in each case as the first derivative maximum of the CD denaturation curves. All the melting and first derivative (dCD_222_/dT vs. T) curves were averaged over 3 scans. Error bars are means ± SD of 3 independent experiments.

### 2.4. CD Spectra Deconvolution

For the deconvolution of the circular dichroism spectra, CD (mdeg) and wavelength (nm) data were given as input to the software CD3 (http://lucianoabriata.altervista.org/jsinscience/cd/cd3.html, accessed on 14 December 2021) [44,45]. Only data corresponding to positive coefficient values were selected for the protein structure analysis choosing the following option: ‘Fit alpha beta coil’.

### 2.5. Fluorescence Studies

Fluorescence spectra were recorded using a spectrofluorometer FluoroMax-4 (Horiba Scientific) at 25 °C. BSA samples were prepared in 1X PBS buffer (137 mM NaCl, 2.7 mM KCl, 10 mM Na_2_HPO_4_, and 1.8 mM KH_2_PO_4_, pH = 7.4) at a concentration of 120 nM and were excited at λ = 280 nm (λ_em_ = 295–470 nm). Subsequently, different aliquots of BDF1 and BF1 (Conc. of the stock solution = 24 µM in DMSO) were added to BSA in order to explore a range of concentrations from 30 to 480 nM. The same titration was repeated adding the same volume of solvent (DMSO). The slit widths were set to 5 nm for both excitation and emission. All the spectra were recorded in duplicate.

### 2.6. Molecular Docking and in Silico Protein–Protein and Protein–Ligand Interaction Analysis

Molecular docking analysis, a methodology widely employed in drug discovery [46,47,48,49,50], in the specific case of the interaction of BF1 and BDF1 with BSA was performed using PatchDock, a docking program based on ligand–receptor geometric shape complementarity [51,52]. FireDock software was then used for rescoring and refinement thanks to its ability to improve the flexibility and scoring errors typically had during the molecular docking calculations by fast rigid-body docking tools [53]. More in detail, the input for PatchDock consisted of PDB files of BF1 and BDF1 ligands, and BSA. The structure of BSA was obtained from the Protein Data Bank database (PDB; ID: 4f5s); the clustering RMSD (root mean square deviation) was set to 4.0 Å, and the complex type was set as the default type. The top 10 results for both BF1 and BDF1 dockings were transferred to FireDock for refinement. The top-ranked FireDock solutions, according to the contribution of the atomic contact energy (ACE), were chosen for the study of the complexes in analogy to other literature examples [54]. Inter-protein residue–residue contacts for homo-dimeric BSA were predicted by DeepHomo Server (http://huanglab.phys.hust.edu.cn/deephomo/, accessed on 31 December 2021) [55], while the protein–ligand interaction diagrams reported in this work were obtained by PLIP (Protein–Ligand Interaction Profiler, https://plip-tool.biotec.tu-dresden.de/, accessed on 31 December 2021) [56].

### 2.7. Pharmacokinetic Properties

We predicted for BF1 and BDF1 the logarithms of the partition coefficients (cLogP), blood–brain barrier (BBB) permeability, pan-assay interference compounds (PAINS) score, and druggability properties presented in this work and in Supporting Information by SwissADME (http://www.swissadme.ch/index.php, accessed on 14 December 2021).

## 3. Results and Discussion

In order to achieve insights on the molecular interactions of benzofurans with the model protein BSA (bovine serum albumin), we performed both spectroscopic [circular dichroism (CD) and fluorescence] and in silico (molecular docking, ligand-protein analysis) studies as described in the sections below.

### 3.1. CD Binding Studies on BSA in Complex with BF1 and BDF1

**Figure 2:**
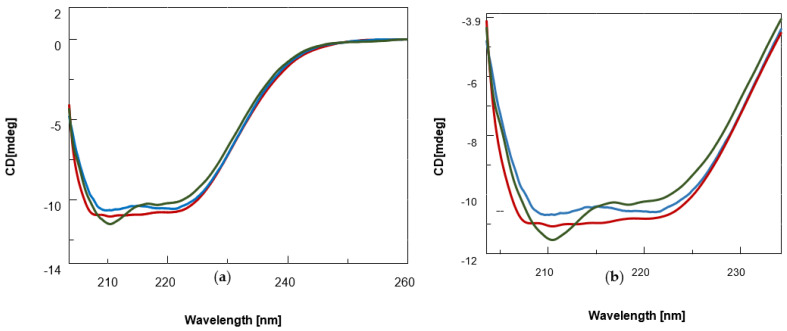
(**a**) CD spectra of BSA (0.12 μM, blue) and its complexes with the benzofuran derivatives (25 nmol) BF1 (dark green) and BDF1 (red) in 90 mM NaCl, 1.8 mM KCl, 6.6 mM Na_2_HPO_4_, 1.2 mM KH_2_PO_4_ (pH = 7.5) at 20 °C. (**b**) Zoomed-in view of the CD bands between 200–235 nm.

It is well known that the BSA secondary structure is mainly dominated by α-helix structures, which account for approximately 60% of its structure, while β–sheet content is less than 10% [57]. Accordingly, in our experiments, the far-UV CD spectra of unliganded BSA exhibited the characteristic features of the typical helical structure of the proteins with two negative bands at 208 and 222 nm (Figure 2, blue). After addition of BF1, the signal intensity at 208 nm was slightly greater than at 222 nm (line dark green), which suggested an increase in β-sheet content in the protein structure as a consequence of the interaction with the ligand as reported in the literature for similar spectral changes [58]. On the other hand, the addition of the benzodifuran BDF1 led to the spectral curve in red (Figure 2), which did not show any predominant band between 208 and 222 nm. These evidences suggest that the secondary structure of BSA underwent slight but significant modifications as consequence of the binding with BF1 and BDF1, with the former being able also to increase slightly the β-sheet content of the albumin structure. To achieve a more quantitative information on this aspect, we then performed a deconvolution of the CD spectra and reported the variations of secondary structure contents of BSA in the absence and presence of an excess of benzofurans, as shown in Table 1.

**Figure 3:**
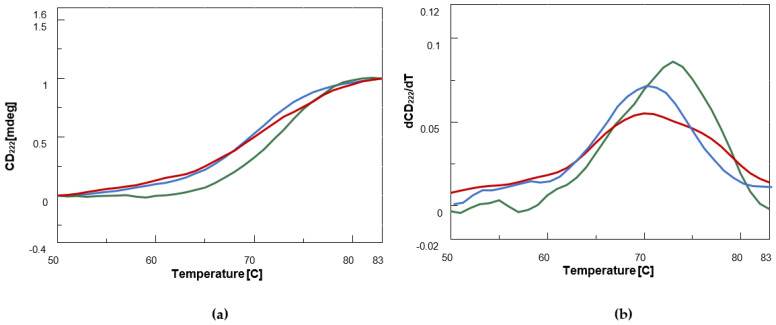
CD thermal denaturation curves [CD_222_ (mdeg) vs. T (°C)] (**a**) and first derivatives vs. T (**b**) plots for BSA (0.12 μM, blue) and its complexes with the benzofuran derivatives (25 nmol) BF1 (green) and BDF1 (red) in 90 mM NaCl, 1.8 mM KCl, 6.6 mM Na_2_HPO_4_, 1.2 mM KH_2_PO_4_ (pH = 7.5) at 20 °C.

According to this table, BDF1 provoked only minor secondary structure changes of BSA, while a certain increase in β-sheet (+5.70%) was observed in the case of the complex of the albumin with BF1 confirming our initial analysis and the literature considerations on the increase in the 208 nm/222 nm band ratio [58]. To investigate the effect of the two classes of benzofurans on protein stability, we recorded CD denaturation curves monitoring the CD values at 222 nm vs. temperature (Figure 3a).

By examining the first derivative maximum of the melting curves, we could demonstrate that while BDF1 led to a slight destabilization (by less than 1 °C) of BSA structure, BF1 increased melting temperature (T_m_) by about 3 °C (Figure 3b, Table 2).

Taken together, the CD binding and melting studies suggested that only BF1 increases BSA β-sheet content and thermal stability, which are both features related to BSA oligomerization. Conversely, BDF1 does not significantly affect the structure elements rate in the albumin and does not provoke any thermal stabilization. This experimental evidence could be explained assuming that in binding to monomeric BSA, BDF1 prevents its aggregation, and/or that its interaction with dimer albumin does affect protein dimerization favouring dissociation into monomeric BSA, in analogy to other literature reports [22].

### 3.2. Fluorescence Studies

**Figure 4:**
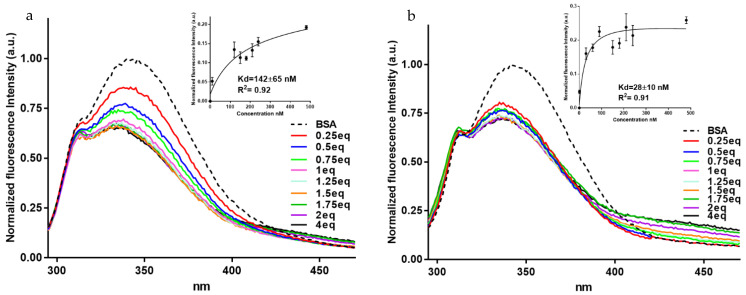
Fluorescence titrations of BSA (120 nM) with (**a**) BDF1 and (**b**) BF1 with ligand concentrations from 30 to 480 nM. Insets: changes in the normalized fluorescence intensity as a function of ligand concentrations (nM) for the titrations of BSA with BDF1 and BF1, after the DMSO background subtraction. k_D_ values with standard deviations determined by the fluorescence method are also reported.

Fluorescence spectroscopy was also used by us to confirm BSA complex formation with BF1 and BDF1 and to have more quantitative information on the affinities of the ligands for the protein target. The fluorescence method is a sensitive tool to study the interactions between proteins such as BSA and small molecules. The molecular recognition of BSA by small molecules mainly determines a static quenching, with the fluorescence being quenched due to the formation of complexes between the fluorophore and quenchers in the ground state [59]. In general, proteins contain three fluorophores, i.e., the amino acids L-tryptophan, L-tyrosine and L-phenylalanine. Due to the low quantum yield of L-phenylalanine and almost quenched characteristics of L-tyrosine, the intrinsic protein fluorescence occurs mainly due to the L-tryptophan. BSA possesses two L-tryptophan residues, Trp-134 and Trp-213. While this latter is situated within a hydrophobic binding pocket of the protein, Trp-134 is found on the surface in the hydrophilic region of the molecule [60]. In our experiment, when exciting at 280 nm, the BSA showed a strong emission band at 347 nm. Interestingly, both compounds led to albumin fluorescence quenching (Figure 4a,b) and their interaction was associated with a good affinity with dissociation rates (k_D_) in the nanomolar range, with BF1 showing a higher affinity than BDF1 (k_D_ = 28.4 ± 10.1 nM vs. 142.4 ± 64.6 nM, insets of Figure 4a,b). More in detail, the BSA emission band was monitored after adding the ligands. Successive additions of benzofurans to BSA led to significant changes in the fluorescence emission. The fluorescence intensity of BSA decreased and blue-shifted by about 10 nm with the addition of increasing amounts of both ligands. The fluorescence quenching, along with the blue shifts, are indicative of the formation of a complex between the BSA and ligands. Additionally, the formation of the complex between the albumin and both benzofurans is indicative of changes of the L-tryptophan environment. After subtracting the DMSO (dimethyl sulfoxide) emission spectrum (as background signal), the fluorescence values were plotted as functions of the concentrations, and from the data fitting it was possible to calculate the apparent k_D_, as reported in the insets of Figure 4a,b.

### 3.3. In Silico Studies on the Benzofuran/BSA Complexes

**Figure 5:**
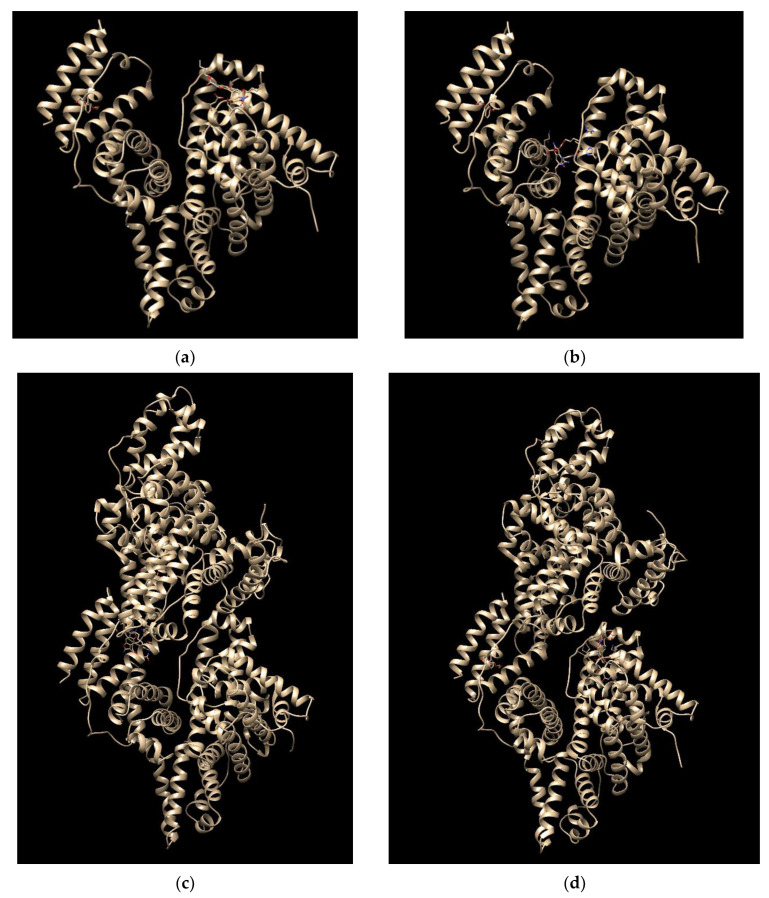
The docked structure of the BDF1/BSA (**a**,**c**) and BF1/BSA (**b**,**d**) complexes with the monomer (**a**,**b** PDB ID: 4f5s, chain: A) and dimer (**c**,**d** PDB ID: 4f5s, chains: A,B) protein targets.

Aiming at giving a possible interpretation of the binding evidence described in the previous sections, we performed a molecular docking study. First, the PatchDock program was used for blind molecular dockings between BSA and BF1 and BDF1. The top-10 solutions from the results of this shape complementarity-based docking methodology [51,52], which produced near native conformation of BSA–ligand complexes (Figure 5), were screened for further refinement and rescoring by using FireDock program to improve the flexibility and scoring errors obtained from the previous rigid dockings [53]. Final results of dockings of BDF1 and BF1 with BSA are presented in Table 3 and Figure 5.

**Figure 6:**
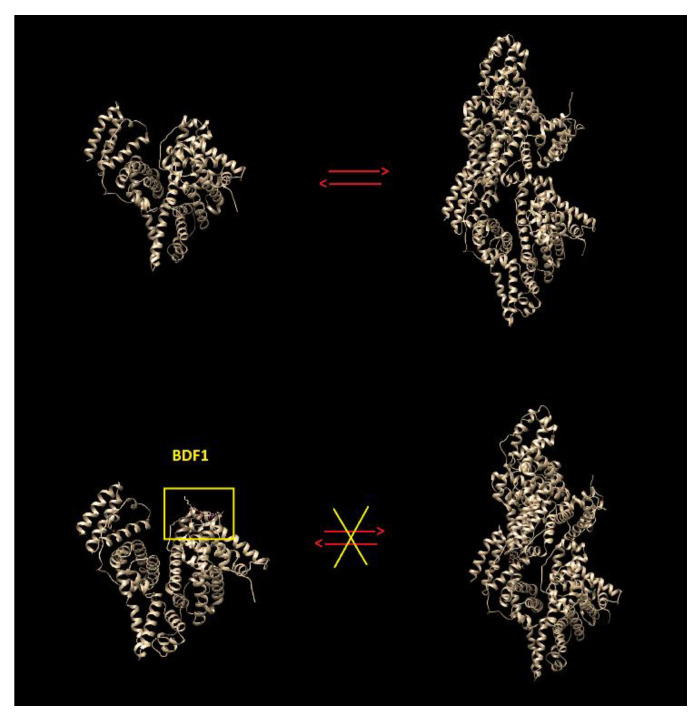
Schematic representation of the monomer—dimer equilibrium of BSA in both its unliganded form and complex with BDF1. Note how BDF1 binds to BSA onto its surface in a region needed for BSA dimerization.

When we docked BDF1 to monomeric and dimeric BSA, an external interaction was predicted onto monomeric BSA surface (Figure 5a) with the main interaction being predicted for the residue Glu-82 (hydrogen bonding, Figure S1, Supplementary data), while an inter-monomer binding of the ligand was observed for the docking with the dimeric BSA (Figure 5c). Global energies for the best docking models were −40.05 and −35.53 kj/mol with atomic contact energies of −6.94 kj/mol and −6.36 kj/mol for monomeric and dimeric BSA, respectively (Table 3). As for the binding of BF1 with BSA, a predicted interaction in the interior of monomeric BSA (Figure 5b) involving numerous residues including Leu-189, Ile-455 (hydrophobic interactions), Glu-424, Ser-428, and Lys-431 (hydrogen bonding, Figure S1 Supplementary data), was clearly revealed by our computational studies. Global and atomic contact energies (−44.20 and −36.78 kj/mol and −11.16 kj/mol and −10.77 kj/mol for monomeric and dimeric albumin, respectively, Table 3) were lower than those found for BDF1, revealing, thus, a higher BF1 affinity for BSA than BDF1, in analogy to the trend that we experimentally found by fluorescence (Figure 4). Interestingly, when docked to dimeric BSA (Figure 5c,d), both benzofurans show a lower predicted affinity for the protein target, and BF1 binds the inner region of one of the two monomers (Figure 5d) involving only residues of chain A (Figure S2, Supplementary data), while BDF1 was predicted to bind at the interface between the two BSA monomers (Figure 5c) with interactions with residues from both A and B chains (Figure S2, Supplementary data). We hypothesize that BDF1 not only prevents the monomer association in dimers in the equilibrium schematically represented in Figure 6, but in analogy to similar literature reports for ligands of dimer albumins, it may provoke dimer dissociation into BSA monomers [22]. In our prediction, the residue involved in the interaction of BDF1 with BSA monomer, i.e., Glu-82, lies in the vicinity of Glu-97 that emerged from our in silico studies as one of the main residues involved in the process of dimerization of BSA (Figure S3, Supplementary data), thus suggesting that BDF1 binding onto the surface of monomeric BSA prevents its dimerization, in accordance with the experimental evidence described in Section 3.1.

Finally, predicting some pharmacokinetic properties for BF1 and BDF1, we found that the former, slightly less hydrophobic than the latter, could be endowed with a more favourable druglikeness profile than the benzodifuran derivative (Figures S4 and S5 Supplementary data). In this prediction, both lack any unspecific biomolecular interacting tendency (PAINS score: 0 for both compounds, Figures S4 and S5).

## 4. Conclusions

We demonstrated here that benzofuran derivatives can efficiently bind to serum albumins, which is of particular importance in drug delivery applications where these proteins can act as carriers of these bioactive heterocyclic molecules. Moreover, we found that benzofurans differing for the number of fused furan rings, such as the benzomonofuran BF1 and the benzodifuran BDF1, may show different binding properties toward the same protein target. In fact, we found that BF1 binds BSA with higher affinity than BDF1 provoking also a thermal stabilization of the albumin (by about 3 °C) not observed in the case of the benzodifuran. Our combined experimental (CD and fluorescence-based) and in silico (molecular docking) investigation led us to conclude that while benzomonofurans bind BSA in internal pockets (altering the Trp-213 environment, which reflects in the observed fluorescence changes), the larger benzodifuran structure interacts externally with the serum albumin in the vicinity of Trp-134 (with effects on the fluorescence of BSA as experimentally observed by us) and residues such as Glu-97 involved in the BSA dimerization, a process consequently disfavoured by BDF1 but not BF1. The different albumin aggregation tendency determined by the ligands could explain the experimentally found differences in albumin stability and β-sheet content, found in our spectroscopic studies. In conclusion, BF1, a potential anticancer drug previously developed in our group, forms stable complexes (k_D_ = 28.4 ± 10.1 nM) with BSA and could likely be efficiently transported in serum by albumins, which could be used as convenient drug delivery systems, especially in this case. On the other hand, BDF1 seems to bind BSA with still high affinity (k_D_ = 142.4 ± 64.6 nM) but less efficiently than BF1 preventing its oligomerization, and it could be further explored in strategies aiming at contrasting protein aggregation.

## Figures and Tables

**Table 1:** Variations in BSA structure content (%) determined by benzofuran ligands. Please note that even though only minor changes can be detected after ligand binding, BF1 determines an increase in β-sheet more significant than BDF1.

	Δ(BF1-BSA) (%)	Δ(BDF1-BSA) (%)
α	+0.06	−0.79
β	+5.70	+1.00
Random coil	−5.76	−0.21

**Table 2:** Summary table of the melting temperatures (T_m_) and their variations (ΔT_m_) with their respective error bars, with respect to the unliganded protein^1^ for the complexes BF1-BSA and BDF1-BSA.

Compound	T_m_/°C	ΔT/°C = (T_m_ − T_m_BSA)
BF1-BSA	72.9 ± 0.1	+3.1 ± 0.2
BDF1-BSA	69.0 ± 0.2	−0.8 ± 0.1

^1^ T_mBSA_ = 69.8 ± 0.1 °C.

**Table 3:** Docking results for the best poses of BF1 and BDF1 in complex with BSA. All energies are given as kj/mol.

Complex	Global Energy	Attractive VDW *	Repulsive VDW *	ACE **
BF1-BSA(monomer)	−44.20	−23.90	11.00	−11.16
BDF1-BSA(monomer)	−40.05	−26.40	11.37	−6.94
BF1-BSA(dimer)	−36.78	−22.73	17.36	−10.77
BDF1-BSA(dimer)	−35.53	−23.79	11.73	−6.36

* VDW = Van der Walls. ** ACE = Atomic contact energy.

## Data Availability

Not applicable.

## Supplementary Materials

The following supporting information can be downloaded at: https://www.mdpi.com/article/10.3390/biom12020262/s1, Figures S1 and S2: 3D interaction diagrams for ligands/BSA complexes; Figure S3: prediction of residue—residue interactions for homodimeric BSA; Figures S4 and S5: predicted pharmacokinetic properties for BF1 and BDF1.
