# Supplementary material for: Protein Binding of Benzofuran Derivatives: A CD Spectroscopic and In Silico Comparative Study of the Effects of 4-Nitrophenyl Functionalized Benzofurans and Benzodifurans on BSA Protein Structure
Source: Biomolecules. 2022 Feb 5;12(2):262. doi: 10.3390/biom12020262 (PMC8961527; doi:10.3390/biom12020262)
Supplement: Supplementary file 1 [file biomolecules-12-00262-s001.zip › biomolecules-1561105-supplementary.pdf]

Supporting Information

Protein binding of benzofuran derivatives: A CD spectroscopic and in silico comparative study of the effects of 4-nitrophenyl functionalized benzofurans and benzodifurans on BSA protein structure.

Pasqualina Liana Scognamiglio [1†], Caterina Vicidomini [2†], Francesco Fontanella [3], Claudio De Stefano [3], Rosanna Palumbo [2], and Giovanni N. Roviello [2,*]

1 Center for Advanced Biomaterial for Health Care (CABHC), Istituto Italiano di Tecnologia, I-80125 Naples, Italy; pasqualina.scognamiglio@iit.it
2 Istituto di Biostrutture e Bioimmagini IBB-CNR; via Tommaso De Amicis 95, I-80145 Naples, Italy;
caterina.vicidomini@ibb.cnr.it; rosanna.palumbo@cnr.it; giroviel@unina.it
3 Department of Electrical and Information Engineering (DIEI), University of Cassino and Southern Lazio, Cassino, Italy; fontanella@unicas.it; destefano@unicas.it
* Correspondence: giroviel@unina.it; Tel.: +39 3491928417 (G. N. R.)
† These authors contributed equally to this work.

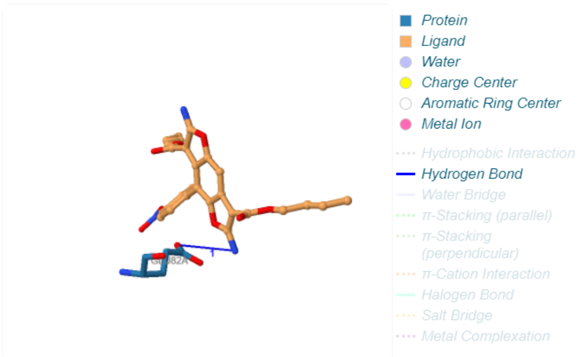

Hydrogen Bonds

Index	Residue	AA	Distance H-A	Distance D-A	Donor Angle	Protein donor?	Side chain	Donor Atom	Acceptor Atom
1	82A	GLU	3.66	4.02	103.99	✗	✓	4865 [Npl]	654 [O2]

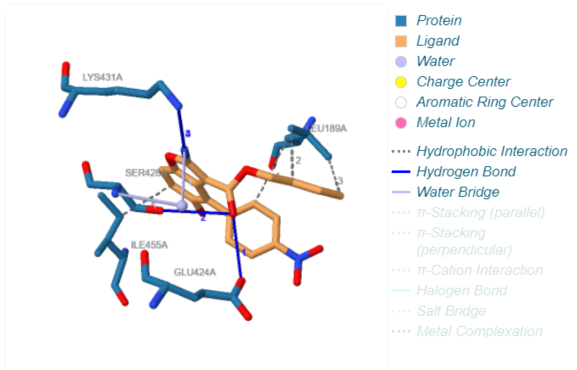

Hydrophobic Interactions

Index	Residue	AA	Distance	Ligand Atom	Protein Atom
1	189A	LEU	3.90	4851	1535
2	189A	LEU	3.05	4848	1537
3	189A	LEU	2.91	4856	1538
4	455A	ILE	3.22	4846	3652

Hydrogen Bonds

Index	Residue	AA	Distance H-A	Distance D-A	Donor Angle	Protein donor?	Side chain	Donor Atom	Acceptor Atom
1	424A	GLU	2.66	3.33	128.77	✓	✓	3416 [O3]	4833 [O2]
2	428A	SER	3.25	3.63	105.62	✓	✓	3446 [O3]	4833 [O2]
3	431A	LYS	2.89	3.45	115.57	✓	✓	3467 [N3]	4836 [Npl]
4	431A	LYS	3.01	3.45	108.66	✗	✓	4836 [Npl]	3467 [N3]

Water Bridges

Index	Residue	AA	Dist. A-W	Dist. D-W	Donor Angle	Water Angle	Protein donor?	Donor Atom	Acceptor Atom	Water Atom
1	428A	SER	3.22	3.46	121.54	92.94	✓	3441 [Nam]	4836 [Npl]	4680

Figure S1. 3D interaction diagrams for BDF1 (up) and BF1 (bottom) in complex with monomeric BSA (PDB ID: 4f5s, chain: A) obtained by PLIP (Protein–Ligand Interaction Profiler, https://plip-tool.biotec.tu-dresden.de/, accessed on 31 December 2021).

Interacting chains: A, B

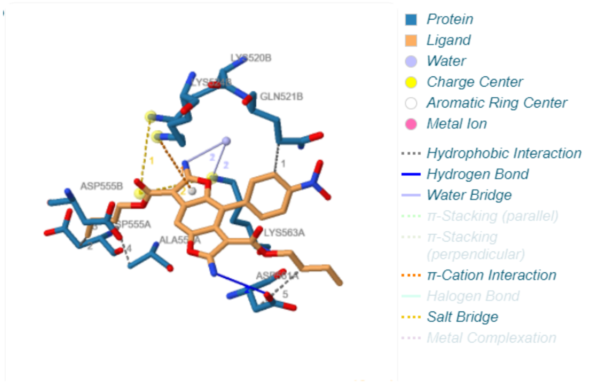

Hydrophobic Interactions

Index	Residue	AA	Distance	Ligand Atom	Protein Atom
1	521B	GLN	3.68	9670	8830
2	555A	ASP	3.90	9644	4452
3	555B	ASP	3.22	9645	9105
4	559A	ALA	3.04	9646	4481
5	561A	ASP	3.86	9659	4491

Hydrogen Bonds

Index	Residue	AA	Distance H-A	Distance D-A	Donor Angle	Protein donor?	Side chain	Donor Atom	Acceptor Atom
1	561A	ASP	2.78	3.64	153.21	✓	✓	4493 [O3]	9664 [Npl]
2	561A	ASP	3.02	3.64	122.52	×	✓	9664 [Npl]	4493 [O3]

Water Bridges

Index	Residue	AA	Dist. A-W	Dist. D-W	Donor Angle	Water Angle	Protein donor?	Donor Atom	Acceptor Atom	Water Atom
1	563A	LYS	2.69	2.97	149.44	134.25	×	9679 [Npl]	4511 [N3]	9451
2	563A	LYS	2.97	2.69	103.44	136.71	✓	4511 [N3]	9679 [Npl]	9451

π-Cation Interactions

Index	Residue	AA	Distance	Offset	Protein charged?	Ligand Group	Ligand Atoms
1	524B	LYS	5.50	1.27	✓	Aromatic	9650, 9651, 9652, 9677, 9678

Salt Bridges

Index	Residue	AA	Distance	Protein positive?	Ligand Group	Ligand Atoms
1	520B	LYS	4.87	✓	Carboxylate	9648, 9680
2	563A	LYS	5.14	✓	Carboxylate	9648, 9680

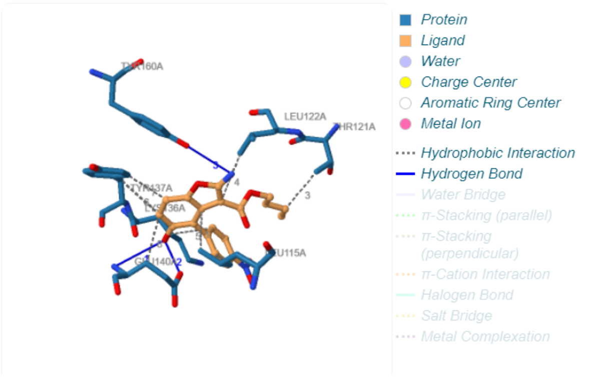

Hydrophobic Interactions

Index	Residue	AA	Distance	Ligand Atom	Protein Atom
1	115A	LEU	3.88	9652	916
2	115A	LEU	2.83	9666	917
3	121A	THR	3.35	9664	963
4	122A	LEU	3.84	9654	970
5	136A	LYS	3.39	9665	1089
6	137A	TYR	3.54	9660	1097
7	137A	TYR	3.13	9659	1099
8	140A	GLU	3.35	9660	1129

Hydrogen Bonds

Index	Residue	AA	Distance H-A	Distance D-A	Donor Angle	Protein donor?	Side chain	Donor Atom	Acceptor Atom
1	140A	GLU	3.53	3.96	108.78	✓	×	1125 [Nam]	9646 [O3]
2	140A	GLU	1.96	2.50	114.78	×	✓	9646 [O3]	1132 [O2]
3	160A	TYR	3.00	3.45	109.14	×	✓	9650 [Npl]	1315 [O3]

Figure S2. 3D interaction diagrams for BDF1 (up) and BF1 (bottom) in complex with dimeric BSA (PDB ID: 4f5s, chains: A,B) obtained by PLIP (Protein–Ligand Interaction Profiler, https://plip-tool.biotec.tu-dresden.de/, accessed on 31 December 2021).

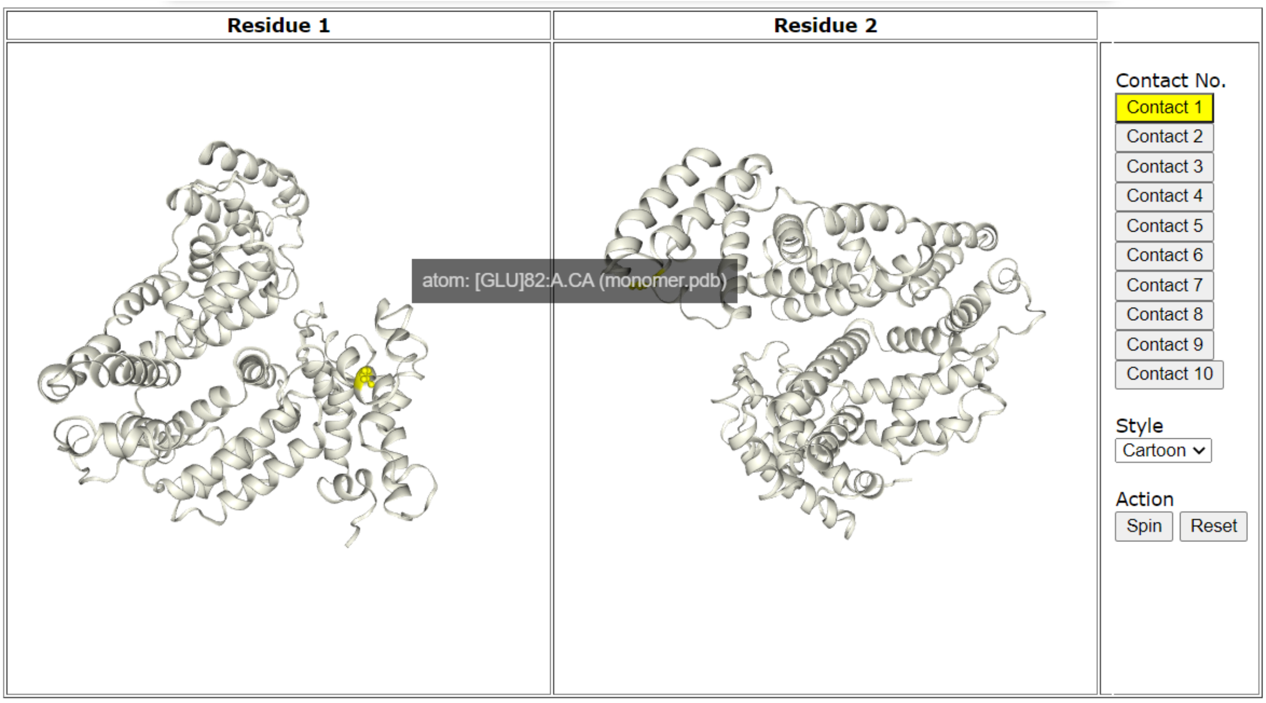

Summary of the top 10 contacts

Rank	1	2	3	4	5	6	7	8	9	10
**Residue 1**	GLU 97	CYS 101	GLU 97	ARG 98	LYS 180	LYS 64	GLU 97	GLU 63	GLU 100	GLU 97
**Residue 2**	HIS 509	HIS 509	PRO 572	HIS 509	GLU 339	GLU 503	LYS 573	PHE 501	HIS 509	GLY 571
**Predicted Score**	0.2149	0.2110	0.2052	0.1996	0.1969	0.1819	0.1795	0.1791	0.1790	0.1787

Figure S3. Inter-protein residue-residue contacts for homo-dimeric BSA as predicted by DeepHomo Server (http://huanglab.phys.hust.edu.cn/deephomo/data/61ce50fad2e29/, accessed on 31 December 2021). Note how Glu-82 lies in the vicinity of one of the main residues (Glu-97) involved in the dimerization of BSA as predicted by DeepHomo Server.

Molecule 1

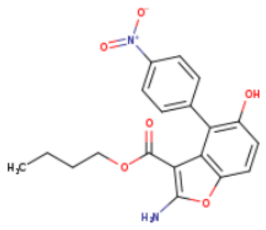

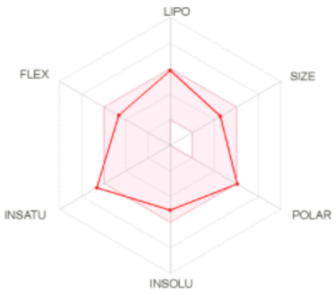

SMILES CCCCOC(=O)c1c(N)oc2c1c(c(cc2)O)c1ccc(cc1)[N+](=O)[O-]

Physicochemical Properties	
Formula	C19H18N2O6
Molecular weight	370.36 g/mol
Num. heavy atoms	27
Num. arom. heavy atoms	15
Fraction Csp3	0.21
Num. rotatable bonds	7
Num. H-bond acceptors	6
Num. H-bond donors	2
Molar Refractivity	102.60
TPSA	131.51 Å²

Lipophilicity	
Log $P_{o/w}$ (iLOGP)	2.21
Log $P_{o/w}$ (XLOGP3)	4.73
Log $P_{o/w}$ (WLOGP)	4.26
Log $P_{o/w}$ (MLOGP)	1.82
Log $P_{o/w}$ (SILICOS-IT)	1.64
Consensus Log $P_{o/w}$	2.93

Water Solubility	
Log *S* (ESOL)	-5.07
Solubility	3.19e-03 mg/ml ; 8.61e-06 mol/l
Class	Moderately soluble
Log *S* (Ali)	-7.22
Solubility	2.23e-05 mg/ml ; 6.01e-08 mol/l
Class	Poorly soluble
Log *S* (SILICOS-IT)	-5.49
Solubility	1.21e-03 mg/ml ; 3.26e-06 mol/l
Class	Moderately soluble

Pharmacokinetics	
GI absorption	Low
BBB permeant	No
P-gp substrate	No
CYP1A2 inhibitor	Yes
CYP2C19 inhibitor	Yes
CYP2C9 inhibitor	Yes
CYP2D6 inhibitor	No
CYP3A4 inhibitor	Yes
Log K_p (skin permeation)	-5.20 cm/s

Druglikeness	
Lipinski	Yes; 0 violation
Ghose	Yes
Veber	Yes
Egan	Yes
Muegge	Yes
Bioavailability Score	0.55

Medicinal Chemistry	
PAINS	0 alert
Brenk	2 alerts: nitro_group, oxygen-nitrogen_single_bond
Leadlikeness	No; 2 violations: MW>350, XLOGP3>3.5
Synthetic accessibility	3.52

Figure S4. Pharmacokinetic properties, including partition coefficients (cLogP), blood–brain barrier (BBB) permeability, pan-assay interference compounds (PAINS) score, and other druggability properties, predicted by SwissADME (http://www.swissadme.ch/index.php, accessed on 14 December 2021) for BF1.

Molecule 2

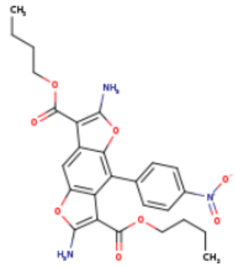

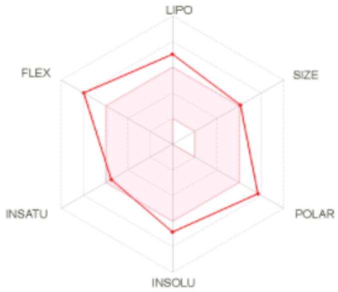

SMILES CCCCOC(=O)c1c(N)oc2c1cc1oc(c(c1c2c1ccc(cc1)[N+](=O)[O-])C(=O)OCCCC)N

Physicochemical Properties	
Formula	C26H27N3O8
Molecular weight	509.51 g/mol
Num. heavy atoms	37
Num. arom. heavy atoms	18
Fraction Csp3	0.31
Num. rotatable bonds	12
Num. H-bond acceptors	8
Num. H-bond donors	2
Molar Refractivity	140.45
TPSA	176.74 Å²

Lipophilicity	
Log $P_{o/w}$ (iLOGP)	3.83
Log $P_{o/w}$ (XLOGP3)	6.80
Log $P_{o/w}$ (WLOGP)	5.85
Log $P_{o/w}$ (MLOGP)	2.29
Log $P_{o/w}$ (SILICOS-IT)	3.10
Consensus Log $P_{o/w}$	4.37

Water Solubility	
Log *S* (ESOL)	-6.85
Solubility	7.18e-05 mg/ml ; 1.41e-07 mol/l
Class	Poorly soluble
Log *S* (Ali)	-10.32
Solubility	2.44e-08 mg/ml ; 4.80e-11 mol/l
Class	Insoluble
Log *S* (SILICOS-IT)	-7.75
Solubility	9.10e-06 mg/ml ; 1.79e-08 mol/l
Class	Poorly soluble

Pharmacokinetics	
GI absorption	Low
BBB permeant	No
P-gp substrate	No
CYP1A2 inhibitor	No
CYP2C19 inhibitor	Yes
CYP2C9 inhibitor	Yes
CYP2D6 inhibitor	No
CYP3A4 inhibitor	Yes
Log K_p (skin permeation)	-4.58 cm/s

Druglikeness	
Lipinski	No; 2 violations: MW>500, NorO>10
Ghose	No; 3 violations: MW>480, WLOGP>5.6, MR>130
Veber	No; 2 violations: Rotors>10, TPSA>140
Egan	No; 1 violation: TPSA>131.6
Muegge	No; 2 violations: XLOGP3>5, TPSA>150
Bioavailability Score	0.17

Medicinal Chemistry	
PAINS	0 alert
Brenk	3 alerts: more_than_2_esters, nitro_group, oxygen-nitrogen_single_bond
Leadlikeness	No; 3 violations: MW>350, Rotors>7, XLOGP3>3.5
Synthetic accessibility	4.39

Figure S5. Pharmacokinetic properties, including partition coefficients (cLogP), blood–brain barrier (BBB) permeability, pan-assay interference compounds (PAINS) score, and other druggability properties, predicted by SwissADME (http://www.swissadme.ch/index.php, accessed on 14 December 2021) for BDF1.
